# Emerging Technologies for Detecting the Chemical Composition of Plant and Animal Tissues and Their Bioactivities: An Editorial

**DOI:** 10.3390/molecules27092620

**Published:** 2022-04-19

**Authors:** Mostafa Gouda, Yong He, Alaa El-Din Bekhit, Xiaoli Li

**Affiliations:** 1 College of Biosystems Engineering and Food Science, Zhejiang University, 866 Yuhangtang Road, Hangzhou 310058, China; 2 Department of Nutrition & Food Science, National Research Centre, Dokki, Giza 12422, Egypt; 3 Department of Food Sciences, University of Otago, Dunedin 9054, New Zealand; aladin.bekhit@otago.ac.nz

Integrating physical and chemical technologies for the characterization and modification of plants and animal tissues has been used for several decades to improve their detection potency and quality [1]. Scientists have been exploring the scientific basis and mechanism of action of the chemical constituents in biological tissues [1,2]. Additionally, special attention has been paid to investigating the different methods for maximizing the detection efficacy of their bioactivities and understanding the changes in their chemical compositions [3,4,5,6]. As an example, using ultrasound (US) in detecting the chemical composition of the biological tissues and their bioactivities has become an important emerging technologies [7,8]. Bourdeau, et al. [9] developed an acoustic method for visualizing and imaging the microbial cellular chemical composition inside mammalian hosts in vivo. In addition, these methods proved their efficiency in their application as analytical methods. For example, acoustic sensors based on quartz crystal microbalance (QCM) were used to detect tea aroma (e.g., linalool, geraniol, linalool oxide, and Trans-2- hexenal) during its fermentation process [10]. In addition, micro/nano-acoustic biosensors are frequently used to enhance the activity of specific biomolecules such as enzymes for increasing detection sensitivity [11]. These biosensors are based on a unique class of air-filled protein nanostructures called gas vesicles that vibrate in response to US waves. The use of US can easily image deep tissue with high spatiotemporal resolution. For instance, Jiang, et al. [12] used US for the bio-imaging of plant chemical composition by using quantum dots technology for in vitro cell imaging and the in vivo imaging of natural plants. Moreover, Lakshmanan, Jin, Nety, Sawyer, Lee-Gosselin, Malounda, Swift, Maresca and Shapiro [8] used acoustic biosensors for imaging the enzyme activity inside the mouse gastrointestinal tract. The principle of using acoustic-based biosensors is based on coupling the measurement nature (such as analyte adsorption) as a modulation in the physical properties of the acoustic wave (such as US frequency and velocity) that could be correlated with the analyte concentration [11]. Existing molecular biosensors, based on fluorescent emission, have limited utility due to the scattering of light and interference with their phytochemicals’ fluorescents. The use of US can easily image deep tissue with high spatiotemporal resolution. Jiang, Jin and Gui [12] used a US-assisted solvothermal reaction for bio-imaging of plant zinc-ions by using quantum dots technology. The authors suggested that the viability of the technique could be used for in-vitro cell imaging and in vivo imaging of natural plants. 

Furthermore, other emerging technologies have been developed for enhancing analytical measurement efficiency. For instance, Gouda, Chen, Li, Liu and He [1] fabricated an electrochemical method based on single plant cells for tracking the chemical composition and the antioxidant activity during the cultivation process. In addition, recent emerging and chemical-free technologies that are related to the in situ detection of the physicochemical changes in the biological media are one of this Special Issue’s targets. As an example, Gouda, et al. [13] developed a method, based on Raman microspectroscopy and circular dichroism, for tracking the changes in the secondary protein structure of microalgae species and its impact on the physicochemical patterns. Moreover, several studies have documented the efficacy of these technologies for the replacement, enhancement, and improvement of various conventional analytical techniques in detecting animal and plant tissues [6,14,15,16,17,18]. 

Thus, the objective of this Special Issue is to demonstrate the potential of US and other recent physicochemical analytical technologies in providing a comprehensive chemical composition and bioactivity relationship of the different biological and organic chemicals. The topic collection includes, but is not limited to, molecular mechanisms of action of organic and inorganic molecules, especially if giving support to visualization approaches by acoustic-based sensors and biosensors, for example, identifying enzymes’ biomarkers, as well as methodologies to investigate the chemical hazardous pollutants and heavy metals through sonochemistry and other related approaches. Further topic includes food, the environment, biomedicine, biotechnology, and the chemical composition of biosystems. In conclusion, this Special Issue could play an important role in maximizing the phytochemical functionality tracking and detection in the drug discovery and biotechnology fields through a very simple application via sonochemistry, electrochemistry, spectroscopy, and other related applications. 

## Data Availability

Not applicable.

## References

[1] Gouda M., Chen K., Li X., Liu Y., He Y. (2021). Detection of microalgae single-cell antioxidant and electrochemical potentials by gold microelectrode and raman micro-spectroscopy combined with chemometrics. Sens. Actuators B Chem..

[2] Wang Y., Zhou S., Wang M., Liu S., Hu Y., He C., Li P., Wan J.B. (2016). Uhplc/q-tofms-based metabolomics for the characterization of cold and hot properties of chinese materia medica. J. Ethnopharmacol..

[3] Dang Z., Liu X., Wang X., Li M., Jiang Y., Wang X., Yang Z. (2019). Comparative effectiveness and safety of traditional chinese medicine supporting qi and enriching blood for cancer related anemia in patients not receiving chemoradiotherapy: A meta-analysis and systematic review. Drug Des. Dev. Ther..

[4] Gouda M., Sheng L., Aadil R.M., Liu Y., Ma M., Li X., He Y., Munekata P.E.S., Lorenzo J.M. (2021). Interaction of bioactive mono-terpenes with egg yolk on ice cream physicochemical properties. Foods.

[5] Lv J.M., Gouda M., Zhu Y.Y., Ye X.Q., Chen J.C. (2021). Ultrasound-assisted extraction optimization of proanthocyanidins from kiwi (actinidia chinensis) leaves and evaluation of its antioxidant activity. Antioxidants.

[6] Lv J.-M., Gouda M., El-Din Bekhit A., He Y.-K., Ye X.-Q., Chen J.-C. (2022). Identification of novel bioactive proanthocyanidins with potent antioxidant and anti-proliferative activities from kiwifruit leaves. Food Biosci..

[7] Gouda M., El-Din Bekhit A., Tang Y., Huang Y., Huang L., He Y., Li X. (2021). Recent innovations of ultrasound green technology in herbal phytochemistry: A review. Ultrason. Sonochemistry.

[8] Lakshmanan A., Jin Z., Nety S.P., Sawyer D.P., Lee-Gosselin A., Malounda D., Swift M.B., Maresca D., Shapiro M.G. (2020). Publisher correction: Acoustic biosensors for ultrasound imaging of enzyme activity. Nat. Chem. Biol..

[9] Bourdeau R.W., Lee-Gosselin A., Lakshmanan A., Farhadi A., Kumar S.R., Nety S.P., Shapiro M.G. (2018). Acoustic reporter genes for noninvasive imaging of microorganisms in mammalian hosts. Nature.

[10] Sharma P., Ghosh A., Tudu B., Sabhapondit S., Baruah B.D., Tamuly P., Bhattacharyya N., Bandyopadhyay R. (2015). Monitoring the fermentation process of black tea using qcm sensor based electronic nose. Sens. Actuators B Chem..

[11] Fogel R., Limson J., Seshia A.A. (2016). Acoustic biosensors. Essays Biochem.

[12] Jiang X., Jin H., Gui R. (2020). Visual bio-detection and versatile bio-imaging of zinc-ion-coordinated black phosphorus quantum dots with improved stability and bright fluorescence. Biosens. Bioelectron..

[13] Gouda M., Huang Z., Liu Y., He Y., Li X. (2021). Physicochemical impact of bioactive terpenes on the microalgae biomass structural characteristics. Bioresour. Technol..

[14] Chu H., Zhang C., Wang M., Gouda M., Wei X., He Y., Liu Y. (2022). Hyperspectral imaging with shallow convolutional neural networks (scnn) predicts the early herbicide stress in wheat cultivars. J. Hazard. Mater..

[15] Zhao Y., Zhang J., Gouda M., Zhang C., Lin L., Nie P., Ye H., Huang W., Ye Y., Zhou C. (2022). Structure analysis and non-invasive detection of cadmium-phytochelatin2 complexes in plant by deep learning raman spectrum. J. Hazard. Mater..

[16] Rehman K.u., Gouda M., Zaman U., Tahir K., Khan S.U., Saeed S., Khojah E., El-Beltagy A., Zaky A.A., Naeem M. (2022). Optimization of platinum nanoparticles (ptnps) synthesis by acid phosphatase mediated eco-benign combined with photocatalytic and bioactivity assessments. Nanomaterials.

[17] Taha M.F., Abdalla A., ElMasry G., Gouda M., Zhou L., Zhao N., Liang N., Niu Z., Hassanein A., Al-Rejaie S. (2022). Using deep convolutional neural network for image-based diagnosis of nutrient deficiencies in plants grown in aquaponics. Chemosensors.

[18] Zong W., Gouda M., Cai E., Wang R., Xu W., Wu Y., Munekata P.E.S., Lorenzo J.M. (2021). The antioxidant phytochemical schisandrin a promotes neural cell proliferation and differentiation after ischemic brain injury. Molecules.

